# COMMUNICATION BETWEEN OLDER ADULTS AND ESSENTIAL CONTACTS: ISOLATION AND ASSISTIVE AND INTERACTIVE TECHNOLOGIES

**DOI:** 10.1093/geroni/igac059.2187

**Published:** 2022-12-20

**Authors:** Dinesh Karki, Sarah Hubner, Julie Blaskewicz Boron

**Affiliations:** University of Nebraska Omaha, Omaha, Nebraska, United States; University of Nebraska Omaha, Omaha, Nebraska, United States; University of Nebraska, Omaha, Omaha, Nebraska, United States

## Abstract

Essential contacts (ECs), people providing support/engagement to older adults, are vital to their physical and socio-emotional well-being. Research suggests that COVID-19 has significantly affected the connectedness of aging populations and their ECs, limiting interaction and potentially weakening support networks. Assistive and interactive technologies (AITs) may be useful for mitigating disruptions by facilitating communication. The goal of this study was to explore AIT preferences and needs of ECs for connecting with adults aged 60+. Participants completed a Qualtrics survey via Amazon Mechanical Turk. An initial sample (N=580,MAge=44.3±14.3,White=81%) was collected (December 2020-February 2021). A minority sub-sample (N=79,MAge=38.9±11.6,Non-White=87.3%) was subsequently accrued (September-December 2021). Participants were initial/minority-ECs for community-dwelling (initial-CDECs=57%,minority-CDECs=89%) and institutionalized adults (initial-IECs=43%,minority-IECs=11%). Minority respondents overwhelmingly identified as CDECs. Results revealed that recent ,“daily/often” phone-based, video-based, and in-person communication were all, more-frequently reported in minority-ECs compared to the initial-EC sample. Phone-based communication was most-frequent in minority-IECs (61%,versus initial-IECs=49%), but was relatively ubiquitous among all (minority-CDECs=49%,initial-CDECs=43%). Initial-CDECs had the least video-based contact (17%); the remaining groups were relatively similar (minority-CDECs=36%,minority-IECs=33%,initial-IECs=32%). CDECs reported the most in-person communication (minority-CDECs=70%,versus initial-CDECs=49%); IECs reported the least (minority-IECs=44%, versus initial-IECs=14%,). Results suggest that this minority sub-set may communicate more, respectively. This may be influenced by population and data-collection-time differences. Still, CDECs may have maintained better access to in-person communication and relied less-heavily on distanced networks through the pandemic. Ultimately, individual characteristics (i.e., ethnicity) and experiences (i.e., COVID-19) may drive communication patterns and end-user needs; this could inform innovation. Future investigation into these topics is warranted.

